# Treatment-resistant depression and risk of autoimmune diseases: evidence from a population-based cohort and nested case-control study

**DOI:** 10.1038/s41398-023-02383-9

**Published:** 2023-03-03

**Authors:** Vivien Kin Yi Chan, Hao Luo, Sandra Sau Man Chan, Chak Sing Lau, Winnie Wan Yin Yeung, Kuan Peng, Xinning Tong, May Pui San Lam, Ian Chi Kei Wong, Xue Li

**Affiliations:** 1 Centre for Safe Medication Practice and Research, Department of Pharmacology and Pharmacy, Li Ka Shing Faculty of Medicine, The University of Hong Kong, Hong Kong SAR, China; 2 Department of Social Work and Social Administration, Faculty of Social Sciences, The University of Hong Kong, Hong Kong SAR, China; 3 Department of Psychiatry, Faculty of Medicine, The Chinese University of Hong Kong, Hong Kong SAR, China; 4 Department of Medicine, School of Clinical Medicine, Li Ka Shing Faculty of Medicine, The University of Hong Kong, Hong Kong SAR, China; 5 Aston Pharmacy School, Aston University, Birmingham, B4 7ET UK; 6 Laboratory of Data Discovery for Health (D24H), Hong Kong Science Park, Hong Kong SAR, China

**Keywords:** Depression, Scientific community

## Abstract

Recent literature indicates that patients with depression had increased immune activation. We hypothesised that treatment-resistant depression (TRD), an indicator of non-responsive depression with long-term dysregulated inflammation, could be an independent risk factor for subsequent autoimmune diseases. We performed a cohort study and a nested case-control study to examine the association between TRD and risk of autoimmune diseases, and to explore potential sex-specific difference. Using electronic medical records in Hong Kong, we identified 24,576 patients with incident depression between 2014 and 2016 without autoimmune history and followed up from diagnosis to death or December 2020 to identify TRD status and autoimmune incidence. TRD was defined as having at least two antidepressant regimens and the third regimen to confirm previous treatment failures. Based on age, sex and year of depression, we matched TRD patients 1:4 to the non-TRD in the cohort analysis using nearest-neighbour matching, and matched cases and controls 1:10 using incidence density sampling in the nested case-control analysis. We conducted survival analyses and conditional logistic regression respectively for risk estimation, adjusting for medical history. Across the study period, 4349 patients without autoimmune history (17.7%) developed TRD. With 71,163 person-years of follow-up, the cumulative incidence of 22 types of autoimmune diseases among the TRD patients was generally higher than the non-TRD (21.5 vs. 14.4 per 10,000 person-years). Cox model suggested a non-significant association (HR:1.48, 95% CI: 0.99–2.24, *p* = 0.059), whereas conditional logistic model showed a significant association (OR: 1.67, 95% CI: 1.10–2.53, *p* = 0.017) between TRD status and autoimmune diseases. Subgroup analysis showed that the association was significant in organ-specific diseases but not in systemic diseases. Risk magnitudes were generally higher among men compared to women. In conclusion, our findings provide evidence for an increased risk of autoimmune diseases in patients with TRD. Controlling chronic inflammation in hard-to-treat depression might play a role in preventing subsequent autoimmunity.

## Introduction

Approximately 280 million people worldwide live with depression, which is ranked a leading cause of disability and a major contributor to the global disease burden [1, 2]. Antidepressants are important treatment modalities with significantly increasing consumption between 2008 and 2019 [3]. However, more than half of patients fail to respond to their first trial of antidepressant, and a significant proportion still do not reach remission following subsequent therapies [4]; as a result, these patients are known to have treatment-resistant depression (TRD). Compared with responders, patients with TRD often further present a significant fatal and non-fatal burden on society and the healthcare system in the form of excessive premature deaths and utilisation of various types of healthcare resources [5].

The multifactorial nature of depression also stimulates a growing interest in its impact on physical health. Due to the bidirectional crosstalk between the endocrine and immune systems, depression and inflammation often influence each other via the balance of cytokine messengers [6, 7]. Recent literature increasingly indicates the role of depression in predicting subsequent autoimmune diseases owing to the increased immune activation and levels of pro-inflammatory cytokines among depressed individuals [8, 9]. Longitudinal epidemiological studies in the contexts of the United States, United Kingdom and Denmark consistently reported that patients with depression had 1.3- to 2.5-fold higher risk of developing rheumatoid arthritis, inflammatory bowel disease, systemic lupus erythematosus and a group of representative autoimmune diseases [10–13]. Use of antidepressants, on the contrary, appears to have an anti-inflammatory protective effect alongside symptom improvement [14]. It has therefore been speculated that patients with autoimmune diseases, who typically have elevated baseline inflammation, are at risk of treatment refractoriness in depression. Alternatively, patients with TRD could also succumb to a more aberrant inflammatory process and greater risk of autoimmune diseases, compared with treatment-responsive patients [15, 16].

Evidence on the association between TRD and autoimmune diseases was scarce. To date, a population-based study from Israel reported significantly higher rates of allergic diseases and positive antinuclear antibodies among patients with TRD than responders, whilst systemic autoimmune diseases had significantly increased likelihood of TRD [17]. The cross-sectional design of the study, however, precluded the examination of temporal relationship. Another retrospective cohort study conducted in Hungary investigated the clinical characteristics of adult patients with TRD and revealed that TRD was significantly associated with higher probability of autoimmune conditions alongside a series of somatic comorbidities than non-TRD depression [18]. However, the study failed to determine whether the autoimmune conditions occurred before or after incidence of depression. A Danish retrospective cohort study recently studied the association between TRD and groups of medical conditions with temporality consideration and found that TRD patients had two-fold higher risk of subsequent multiple sclerosis in men, which stimulates ongoing attention to the unknown risk of other common autoimmune diseases [19]. The fact that both depression and autoimmune diseases are predominantly found in female patients raises the question of a potentially sex-specific association. To address the limitations of the existing studies and knowledge gap, we aimed to conduct a cohort study and a nested case-control study with more stringent designs to examine the (1) association between TRD and risk of autoimmune diseases and explore (2) any potential sex difference in the association, using the territory-wide longitudinal electronic medical records (EMR) in Hong Kong.

## Methods

### Data source

We used the territory-wide routine EMR database (Clinical Data Analysis and Reporting System) between 1993 and 2020 managed by the Hospital Authority, a statutory body which manages all public hospitals and clinics in Hong Kong and provides publicly funded healthcare services to all eligible residents (>7 million). Real-time updated patient data including demographics, dates of birth and death, dates of service attendances, all-cause diagnoses coded based on the International Classification of Diseases, 9th Revision, Clinical Modification (ICD-9-CM) and prescriptions were comprehensively recorded across the outpatient, inpatient and emergency settings for research and auditing purposes. The database has been used and described in previous epidemiological studies on depression, suicidal attempts and autoimmune diseases in Hong Kong [5, 20–25].

### Study design and participants

We conducted a population-based study using both cohort design and nested case-control design in parallel, with the intention to preserve the advantages and complement the limitations of the other as we consider that cohort studies conventionally have a higher level of evidence, whereas case-control analysis was more appropriate for evaluating rare outcomes. The study period started in January 2014 and ended in December 2020. The cohort consisted of all incident patients aged above 10 years with diagnosis codes for depression (ICD-9-CM codes: 296.2, 296.3, 300.4, 311) between January 2014 and December 2016 without history of diagnosis for depression since 1993, when the database first became available. Patients were excluded if they had history of studied autoimmune diseases before onset of depression, or if they died immediately after cohort entry.

Throughout the study, patients were defined as *treatment-resistant* (exposed) if they had taken at least two antidepressant regimens of adequate duration (same antidepressant or combined therapy of at least 28 days with gaps of no longer than 14 days within regimens, whilst the 28-day duration was the minimum recommended duration to assess treatment responsiveness [26]) and had a third antidepressant regimen to confirm failure of the previous two trials. Patients who did not fulfil the criteria for TRD were considered as non-TRD (unexposed). Onset of outcome was confirmed on the date of the first autoimmune diagnosis in (1) organ-specific diseases including inflammatory bowel diseases, spondyloarthritis, psoriasis, insulin-dependent diabetes mellitus, Hashimoto’s thyroiditis, Graves’ disease, coeliac disease, vitiligo, alopecia areata, pemphigus vulgaris, dermatitis herpetiformis, pernicious anaemia, immune thrombocytopenic purpura, iridocyclitis and pemphigoid, and (2) systemic diseases including systemic lupus erythematosus, rheumatoid arthritis, Sjogren’s disease, systemic sclerosis, polymyositis/dermatomyositis, multiple sclerosis and juvenile arthritis, captured across all settings including outpatient, inpatient and emergency services. List of ICD-9-CM codes to identify the cohort and outcomes is presented in Supplementary Table 1. Using the comorbidity rates reported from a previously similar population-based study, the sample sizes required for data collection were 5403 and 12545 for the analyses in systemic and organ-specific autoimmune diseases, respectively, to achieve an 80% statistical power in the cohort study [17, 27].

### Retrospective cohort analysis

We analysed the prescription trajectory of all cohort participants from the date of depression onset until December 2020 to ascertain their TRD status, and matched patients with TRD 1:4 to the non-TRD based on their year of first depression diagnosis, and then age and sex using nearest-neighbour matching. The follow-up started from the prescription date of the third regimen (index date for patients with TRD) until any censoring events. The same index date was applied to the four non-TRD matches. Patients whose deaths or onset of autoimmune diseases occurred earlier than the assigned index dates were further excluded (Supplementary Fig. 1). We reported the incidence of autoimmune diseases per 10,000 person-years using Poisson distribution. Next, we estimated the hazard ratios (HRs) of any development of autoimmune diseases associated with TRD status, using a multivariable Cox regression model. Censoring events were development of autoimmune diseases (outcome), death and end of study period, whichever came first. We performed a Schoenfeld residual-based test, which showed no violation of proportional hazard assumption. Covariates included any history of physical disorders (obesity, type II diabetes, hypertension, cardiovascular diseases and tumours) and history of psychiatric conditions (attention-deficit hyperactivity disorder, autism, psychosis or schizophrenia, epilepsy, anxiety disorder, personality disorder, substance use disorder, dementia, bipolar disorder, obsessive compulsive disorder and eating disorder) on or before index date. Detailed ICD-9-CM codes for the covariates are reported in the Supplementary Table 1.

### Nested case-control analysis

Within the same overall cohort, we identified cases as patients who had a diagnosis of autoimmune diseases from the date of depression onset until December 2020. Each case was then randomly matched to a maximum of 10 controls by age, sex, and year of first depression diagnosis using incidence density sampling with replacement. The first diagnosis date of autoimmune disease (index date) for cases was assigned the same for their matched controls, who were alive and had no history of autoimmune diseases on the index date. We then determined the TRD status of all patients any time before index date and fitted a conditional logistic regression to estimate odds ratios (ORs) of onset autoimmune diseases associated with TRD. The model was adjusted for the same type of covariates as the cohort study.

### Subgroup and sensitivity analyses

We replicated the analysis by changing the definition of autoimmune diseases to only organ-specific diseases or systemic diseases as outcome (cohort design) or case definition (nested case-control design) and conducted subgroup analysis by sex. Additionally, to address potential competing risk due to premature death, we applied Fine and Grey’s model to assess the subdistribution hazard ratio of autoimmune diseases in the cohort study as a sensitivity analysis. All analyses were performed using R 4.0.3 and cross-checked by two investigators.

## Results

### Patient selection and baseline characteristics

**Fig. 1:**
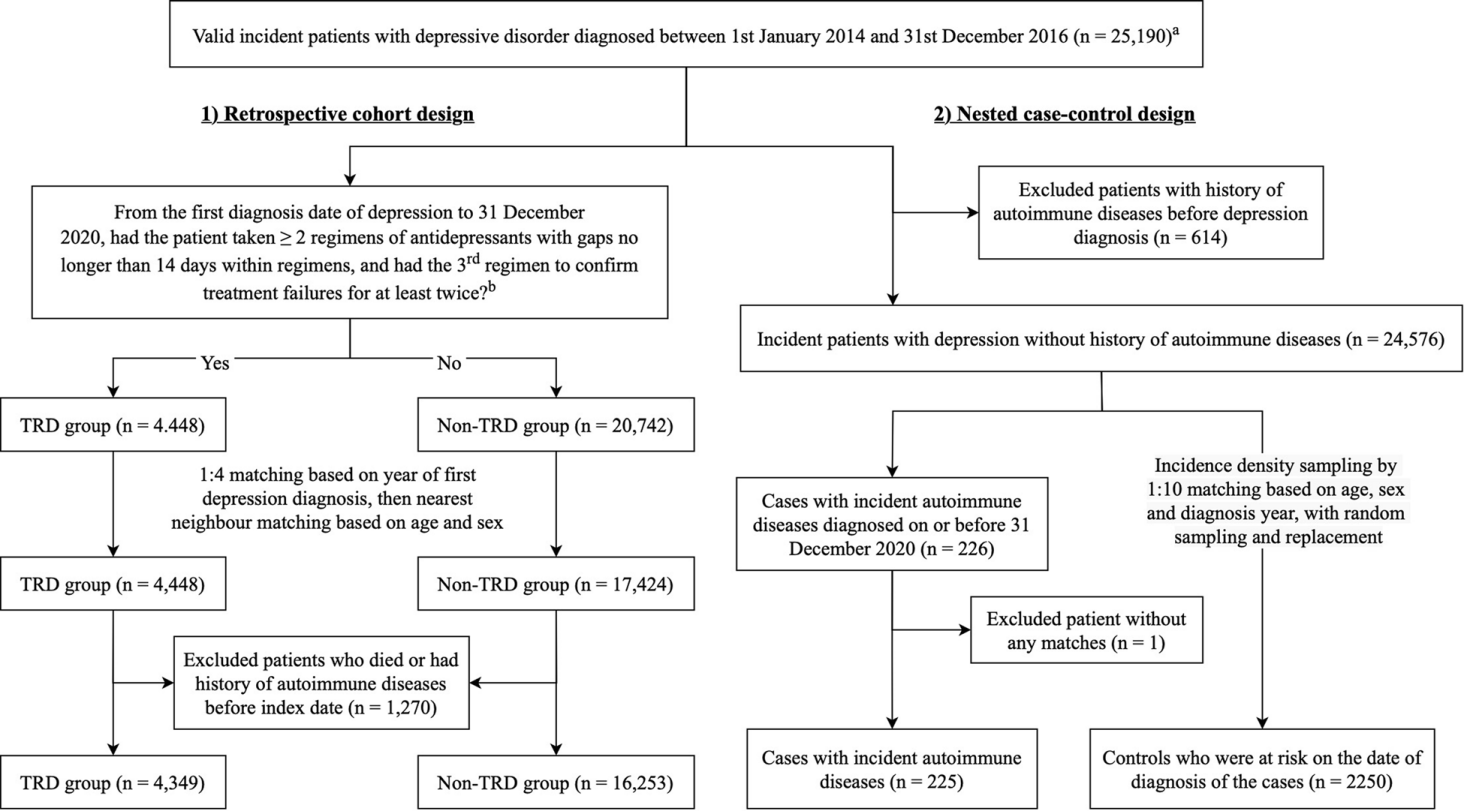
Identification of overall cohort, exposure groups, cases and controls in the retrospective cohort study and nested case-control study. All diagnoses were captured across all settings including outpatient, inpatient and emergency services.^a^Valid incident patients were patients older than 10 years old without records of depression from 1993 to the date of the first recorded depression diagnosis, and who did not die immediately after cohort entry.^b^Prescription records of antidepressants between the date of first depression diagnosis and 31 December 2020 were extracted to define patients' TRD status. Antidepressant treatment regimens could be either monotherapy or combination therapies with antipsychotics and/or mood stabilisers. TRD treatment-resistant depression.

Figure 1 presents the identification process of comparison groups from the overall cohort of incident depression patients. Between 2014 and 2016, we identified a total of 25,190 valid incident patients who were diagnosed with depression for the first time.

Across the follow-up in the cohort study, 4448 patients (17.7%) developed TRD. After matching and excluding patients who died or had history of autoimmune diseases by index date, 4349 patients with TRD and 16,253 patients with non-TRD had similar baseline age, sex distribution and overall history of psychiatric conditions on index date (Table 1). Patients with TRD, however, were significantly more likely to have a history of physical disorders than the patients with non-TRD (24.2% vs. 22.1%, *p* = 0.003). The mean time for patients to develop treatment resistance was 2.0 years (±1.6 years). After the index date, the mean follow-up periods were similar between groups (3.4 ± 1.7 years for TRD; 3.5 ± 1.7 years for the non-TRD group).

**Table 1:** Characteristics of patients with TRD and their matched controls in the cohort study.

	Patients without TRD	Patients with TRD	*p* value	SMD
	*N* = 16253	*N* = 4349		
Age (mean, SD)	46.10 (17.37)	46.64 (17.72)	0.073	0.031
Male (*N*, %)	4157 (25.6)	1104 (25.4)	0.812	0.004
Any history of physical disorders (*N*, %)	3589 (22.1)	1052 (24.2)	0.003^a^	0.050
Obesity (*N*, %)	198 (1.2)	50 (1.1)	0.772	0.006
Type II diabetes (*N*, %)	1052 (6.5)	287 (6.6)	0.790	0.005
Hypertension (*N*, %)	1907 (11.7)	587 (13.5)	0.002^a^	0.053
Cardiovascular diseases (*N*, %)	1749 (10.8)	571 (13.1)	<0.001^a^	0.073
Any tumours (*N*, %)	699 (4.3)	186 (4.3)	0.979	0.001
Any history of psychiatric conditions (*N*, %)	2838 (17.5)	761 (17.5)	0.973	0.001
ADHD (*N*, %)	57 (0.4)	10 (0.2)	0.275	0.022
Autism (*N*, %)	12 (0.1)	6 (0.1)	0.326	0.020
Psychosis (*N*, %)	853 (5.2)	154 (3.5)	<0.001^a^	0.083
Epilepsy (*N*, %)	193 (1.2)	49 (1.1)	0.802	0.006
Anxiety disorder (*N*, %)	787 (4.8)	248 (5.7)	0.023^a^	0.039
Personality disorder (*N*, %)	289 (1.8)	90 (2.1)	0.228	0.021
Substance use disorder (*N*, %)	609 (3.7)	182 (4.2)	0.197	0.022
Dementia (*N*, %)	162 (1.0)	71 (1.6)	0.001^a^	0.056
Bipolar disorder (*N*, %)	191 (1.2)	32 (0.7)	0.016^a^	0.045
OCD (*N*, %)	96 (0.6)	28 (0.6)	0.770	0.007
Eating disorder (*N*, %)	43 (0.3)	12 (0.3)	1.000	0.002
Follow-up time (mean days, SD)	1265 (606)	1250 (621)	0.161	0.024

*ADHD* Attention-deficit Hyperactivity Disorder, *OCD* Obsessive-compulsive disorder, *SD* Standard deviation, *SMD* Standardised mean difference, *TRD* Treatment-resistant depression.

^a^Significant at 0.05 between TRD and non-TRD groups using Chi-square or Fisher’s exact tests.

In the overall cohort of the nested case-control study, we excluded 614 patients (2.4%) who had a history of autoimmune diseases prior to depression. Among the remaining 24,576 patients, 226 patients (0.9%) had developed autoimmune diseases by December 2020 (cases). Of which, 225 had eligible matches of 2250 controls. There were no marked differences between the cases and controls in baseline age, sex distribution, history of physical and psychiatric conditions and depression duration on index date (Table 2). Cases had a significantly higher proportion of TRD than the controls (14.2% vs. 9.4%, *p* = 0.029).

**Table 2:** Characteristics of cases and controls in the nested case-control study.

	Without autoimmune disease development	With autoimmune disease development	*p* value	SMD
	Controls, *N* = 2250	Cases, *N* = 225		
Age (mean, SD)	53.65 (18.75)	53.65 (18.75)	1.000	<0.001
Male (*N*, %)	520 (23.1)	52 (23.1)	1.000	<0.001
Any history of physical disorders (*N*, %)	690 (30.7)	69 (30.7)	1.000	<0.001
Obesity (*N*, %)	28 (1.2)	5 (2.2)	0.361	0.075
Type II diabetes (*N*, %)	196 (8.7)	23 (10.2)	0.524	0.052
Hypertension (*N*, %)	396 (17.6)	31 (13.8)	0.176	0.105
Any cardiovascular diseases (*N*, %)	352 (15.6)	34 (15.1)	0.909	0.015
Any tumours (*N*, %)	135 (6.0)	16 (7.1)	0.605	0.045
Any history of psychiatric conditions (*N*, %)	404 (18.0)	40 (17.8)	1.000	0.005
ADHD (*N*, %)	8 (0.4)	1 (0.4)	1.000	0.014
Autism (*N*, %)	2 (0.1)	1 (0.4)	0.648	0.069
Psychosis (*N*, %)	129 (5.7)	13 (5.9)	1.000	0.005
Epilepsy (*N*, %)	34 (1.5)	2 (0.9)	0.652	0.057
Anxiety disorder (*N*, %)	135 (6.0)	14 (6.2)	1.000	0.009
Personality disorder (*N*, %)	34 (1.5)	2 (0.9)	0.652	0.057
Substance use disorder (*N*, %)	70 (3.1)	6 (2.7)	0.868	0.027
Dementia (*N*, %)	35 (1.6)	6 (2.7)	0.331	0.077
Bipolar disorder (*N*, %)	19 (0.8)	4 (1.8)	0.305	0.082
OCD (*N*, %)	9 (0.4)	1 (0.4)	1.000	0.007
Eating disorder (*N*, %)	5 (0.2)	1 (0.4)	1.000	0.039
Depression duration (mean days, SD)	882 (670)	877 (674)	0.916	0.007
TRD development (*N*, %)	212 (9.4)	32 (14.2)	0.029^a^	0.149

*ADHD* Attention-deficit Hyperactivity Disorder, *OCD* Obsessive-compulsive disorder, *SD* Standard deviation, *SMD* Standardised mean difference, *TRD* Treatment-resistant depression.

^a^Significant at 0.05 between TRD and non-TRD groups using Chi-square or Fisher’s exact tests.

### Incidence of autoimmune diseases

**Fig. 2:**
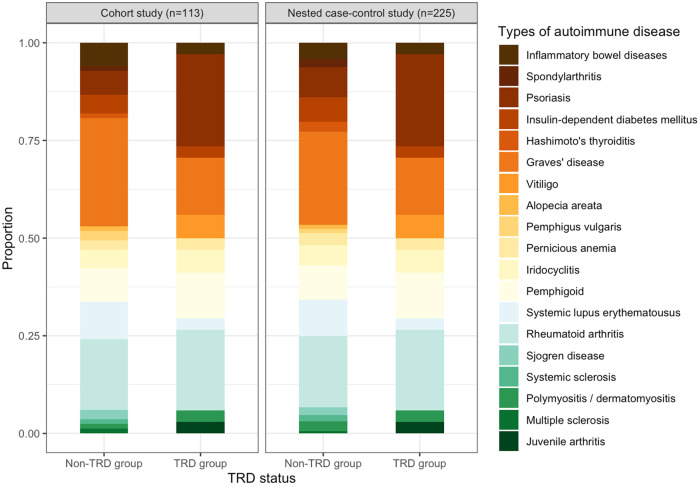
Frequency of occurrence of individual autoimmune diseases during follow-up in the cohort and nested case-control study. ^a^Celiac disease, dermatitis herpetiformis and immune thrombocytopenic purpura were excluded from the chart owing to absence of case during follow-up. TRD treatment-resistant depression.

**Fig. 3:**
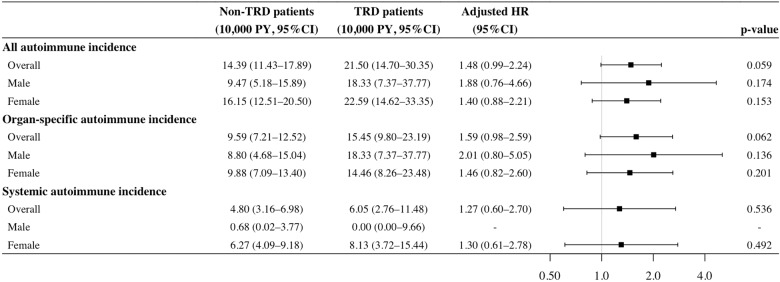
Incidence and adjusted hazard ratios of autoimmune disease development among patients with and without TRD in the cohort study. *Significant at 0.05 in multivariable Cox regression. Multivariable Cox models adjusted for binary indicators of history of physical disorders and history of psychiatric conditions. Analysis was not performed for systemic autoimmune incidence in male patients owing to lack of patients with both TRD and systemic autoimmune diseases. CI confidence interval, HR hazard ratio, PY patient-years, TRD treatment-resistant depression.

In 71,163 person-years of follow-up in the cohort study, 113 patients developed autoimmune diseases [15.9 (95% CI: 13.1–19.1) per 10,000 person-years], including 77 diagnosed with organ-specific diseases and 36 with systemic diseases. In the cohort analysis, the most frequently developed types of autoimmune diseases were Graves’ disease (TRD: 15.6%, non-TRD: 28.4%), rheumatoid arthritis (TRD: 21.9%, non-TRD: 18.5%) and psoriasis (TRD: 25.0%, non-TRD: 6.2%). A similar pattern of autoimmune incidence was found in the nested case-control analysis (Fig. 2). The cumulative incidence of autoimmune diseases in the TRD group was generally higher at 21.5 (95%CI: 14.7–30.4) per 10,000 person-years compared with the non-TRD group [14.4 (95% CI: 11.4–17.9) per 10,000 person-years], and similar trends were observed regardless of sex and type of autoimmune diseases (organ-specific/systemic) (Fig. 3). 75.2% of autoimmune diseases occurred within three years after the index date, with mean onset time of 1.9 years (±1.5 years) and 2.1 years (±1.5 years) in the TRD and non-TRD groups (Supplementary Fig. 2).

### Association between TRD and autoimmune diseases

**Fig. 4:**
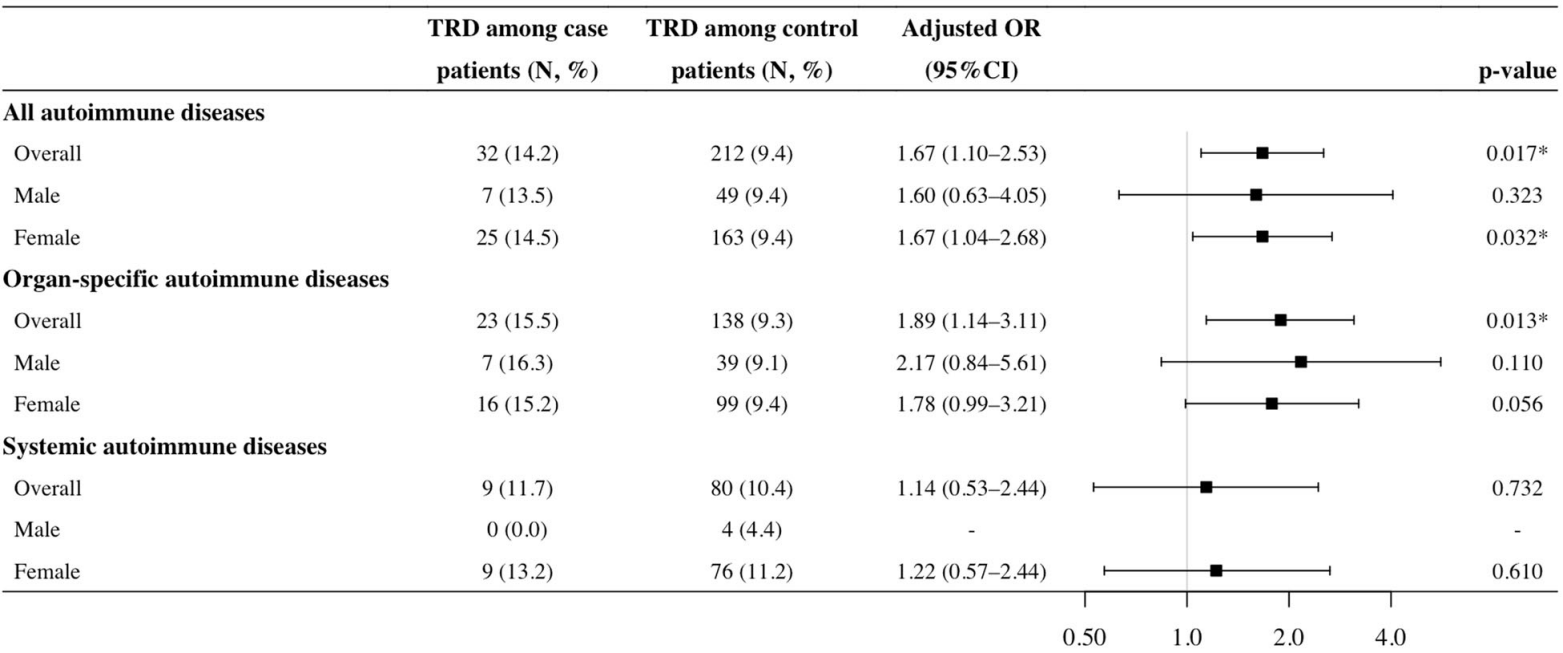
Adjusted odds ratios of autoimmune disease development among patients with and without TRD in the nested case-control study. *Significant at 0.05 in univariable and multivariable conditional logistic regression. Multivariable condition logistic models adjusted for binary indicators of history of physical disorders and history of psychiatric conditions. Analysis was not performed for the systemic autoimmune disease development in male patients owing to lack of case patients with TRD. CI confidence interval, OR odds ratio, TRD treatment-resistant depression.

Figures 3, 4 detail the results of multivariable regression models in the cohort and nested case-control study designs. Grouping all autoimmune diseases, Cox model in the cohort study showed that the adjusted HR of TRD status was 1.48 (95% CI: 0.99–2.24) without statistical significance (*p* = 0.059), whilst the conditional logistic model in the nested case-control study showed a significant association between TRD and risk of autoimmune diseases with an adjusted OR of 1.67 (95% CI: 1.10–2.53, *p* = 0.017). Similar results were obtained when the study outcome was stratified into organ-specific autoimmune diseases only, with an adjusted HR of 1.59 (95% CI: 0.98–2.59, *p* = 0.062) and an adjusted OR of 1.89 (95% CI: 1.14–3.11, *p* = 0.013). The association between TRD status and systemic autoimmune diseases was not significant in the analyses using both study designs.

In the subgroup analysis, the association between TRD status and risk of autoimmune diseases was significant only in women with an adjusted OR of 1.67 (95% CI: 1.04–2.68, *p* = 0.032) in the nested case-control study. Despite non-significant model results, the effect magnitudes were generally higher among men in the association between TRD status and risk of overall autoimmune diseases in the cohort study (adjusted HR: 1.88 vs. 1.40), and in the association between TRD status and risk of organ-specific diseases in both study designs (adjusted HR: 2.01 vs. 1.46; adjusted OR: 2.17 vs. 1.78), compared with women.

In the sensitivity analysis using the Fine and Grey model in the cohort study, the HR of TRD status towards risk of overall autoimmune diseases was 1.47 (95% CI: 0.98–2.21, *p* = 0.065), the magnitude and direction of which were consistent with that in the main analysis, after adjusting for competing risk due to premature death.

## Discussion

Using two study designs based on a territory-wide longitudinal EMR database, the current study presents an attempt to delineate the association between antidepressant resistance and development of autoimmune diseases with consideration of temporal causality. Our findings provide support to the hypothesis that TRD status could be a risk predictor of developing autoimmune diseases, with an adjusted HR of 1.48 (95% CI: 0.99–2.24) at marginal significance in the cohort study and a significant adjusted OR of 1.67 (95% CI: 1.10–2.53) in the nested case-control study. We also identified that the increased risk more likely stemmed from organ-specific autoimmune diseases than systemic autoimmune diseases. The magnitude of risk among men appeared to be generally higher than in women in the studied associations from both study designs and consistent across subtypes of autoimmune diseases.

Triangulation is the practice of increasing reliability of answers to epidemiological questions via integrating results from different study approaches, in which the approaches possess distinct and unrelated assumptions and potential bias [28]. In our study, we designed both a cohort analysis and a case-control analysis “nested” within a common cohort to improve causal inference. The results from both approaches point towards the same conclusion with a similar risk magnitude, which strengthens the reliability of our findings [28]. It is possible to explain the association between depression or TRD and risk of autoimmune diseases in view of the biological mechanism, by which chronic stress could disturb signalling pathways in the sympathetic nervous system and hypothalamic-pituitary-adrenal axis. As the pathways are also involved in the regulation of immune functioning via cytokine activities, sustained stimulation with depressive symptoms may dysregulate the immune system and subsequently lead to autoimmunity [29–31]. Previous studies documented that patients with depression had elevated levels of pro-inflammatory markers, such as C-reactive protein (CRP), interleukin-6 (IL-6) and tumour necrosis factor alpha (TNF-α), which were also manifested in the cytokine profiles of patients with organ-specific and/or systemic autoimmunity [8, 9, 32–35]. Introduction of pro-inflammatory agents, such as interferon-alpha, could induce mild-to-moderate depressive symptoms, whilst antidepressant treatments were associated with decreased production of pro-inflammatory cytokines including IL-6 and TNF-α among responders [8, 36, 37]. In line with the inflammation theory, lack of response towards antidepressants may logically predispose non-responders to a higher probability of dysregulated baseline inflammation and cytokine dynamics owing to the protective effect of antidepressants against inflammation.

Our main findings were largely consistent with current observational studies. Prior to this work, a Hungarian retrospective cohort study analysed six types of somatic comorbidities and reported 22% increase in odds of autoimmune diseases among patients with TRD, despite the inability to determine whether TRD development preceded onset of comorbidities [18]. A cross-sectional study in Israel later reported that patients with TRD had 52% increase in odds of allergic and autoimmune diseases, compared with treatment-responsive patients [17]. The study however reported significant findings in only the pooled group of systemic autoimmune diseases but not organ-specific autoimmune diseases, although the incidence was increased among both TRD and non-TRD depressed patients compared with non-depressed individuals. In contrast, we identified a significant association between TRD status and organ-specific diseases but not systemic diseases. With similar nosologies for organ-specific and systemic autoimmunity, the discrepancy is not entirely clear but may have originated from ethnic or methodological differences, especially given the possible presence of a bidirectional relationship between depression and autoimmunity in a cross-sectional design. In our study, the increased risk of autoimmune diseases was more likely contributed by organ-specific diseases, which may be related to distinct underlying mechanisms of pathogenesis between organ-specific and systemic diseases [38], or alternatively, a sample-size-driven phenomenon due to more types of disease in the organ-specific category. Future studies with larger sample size and event number for systemic diseases will help to differentiate the reasons.

As the patients with depression and autoimmune diseases in our study are predominantly women, it is reasonable that a significant association was observed only among women. However, the risk magnitudes of association appeared to be higher among men instead across study designs and outcome types. Despite non-significance, the observed trend was consistent with previous studies which found that the increased CRP was stronger in male patients with depression compared with female patients [39, 40]. In addition, a previous Danish retrospective cohort study which similarly explored sex-specific difference with temporality consideration also identified an increased risk of multiple sclerosis associated with TRD only in male patients [19]. A possible hypothesis was given by the link between inflammation and the dysregulation of hormonal systems, in which estrogen may exert a protective effect given its anti-inflammatory properties [41, 42]. Over the years, there has been rising awareness of sex-specific disparities in medical research, with the aim to identify health modifiers and vulnerabilities to inform intervention efforts [43]. Although it was not possible to explain the mechanism based on our study, further laboratory-based studies capable of elucidating the potential sex-specific mechanism is important in the identification of vulnerabilities.

Our findings reported herein underpin several implications with regards to understanding and managing hard-to-treat depression. Alongside existing etiological evidence, our study findings adds to support of the key role that inflammation plays in the pathogenesis of depression and treatment resistance, which renders ongoing exploration of anti-inflammatory agents and cytokine inhibitors as antidepressants valuable for patients without satisfactory treatment responses [8]. In the TRD population, clinical awareness should be raised to manage treatment refractoriness early and detect subsequent development of autoimmune diseases, followed by timely intervention to prevent disease progression, such that potential excessive burdens in healthcare resources jointly arising from multifaceted mental and somatic consequences can be avoided. The predictive phenomenon of TRD for autoimmunity also necessitates further research on the causal link between the two conditions. Although our cohort already excluded patients with autoimmune disease history prior to follow-up, it was possible that patients with heightened baseline pro-inflammatory cytokine release may predispose both antidepressant resistance and autoimmunity, whilst hard-to-treat depression manifested before the development of autoimmune diseases. Future mechanistic studies would help dissect the relationship of depression, or treatment resistance, with chronic inflammation- and immune-related disorders, which would be valuable to help determine if medications in autoimmune diseases should be repurposed for depression and reduce the use of antidepressants.

There were also several limitations to our study. First, similar with other studies utilising nation-wide EMR as the data source, we lacked detailed psychometric measurements and clinical information, such as symptom severity or reasons for switching of drug regimens and dosage adjustment; it was thus challenging to determine whether patients stopped or switched medications due to inadequate initial dosing, atypical pharmacokinetics, adverse events, medication compliance or genuine lack of treatment responses. Given the absence of a universal definition of TRD, pseudo-resistance could not be entirely ruled out. Second, despite the higher evidence level of cohort study, the index dates manually assigned to the non-TRD patients may have occurred late if their matched TRD patients received the third regimen near the later stage of follow-up, therefore yielding a reduced number of events following a shorter observation period. The risk comparison using the cohort study design therefore presents a more conservative estimate. To address this, we performed the nested case-control analysis in parallel to maximise the number of observable events since the index dates were defined differently, with the study design less prone to selection bias compared with a conventional case-control study [44]. Third, patients with TRD may have been granted more intense care and visits with increased chance of detecting onset of autoimmune diseases early. Last, although we benchmarked the list of studied autoimmune diseases with existing literature, misclassification was still possible. For instance, systemic lupus erythematosus may have been documented in cases where patients only had lupus nephritis. However, differential bias across exposure groups in the cohort analysis and cases and controls in the nested case-control analysis was unlikely.

## Conclusion

We found increased risk of autoimmune diseases in patients with TRD compared with patients who responded to antidepressants. Inflammation control in hard-to-treat depression might play an important role in the prevention of subsequent autoimmune conditions. Association of sex-specific disparities deserves further mechanistic investigation.

## Supplementary information

Supplementary legend

Supplementary materials

## Data Availability

We are unable to directly share the aggregated data since the data custodian, Hospital Authority who manages the Clinical Data Analysis and Reporting System (CDARS), had not given permission. Alternatively, CDARS data can be directly requested via the Hospital Authority Data Sharing Portal for research purpose.

## Code availability

All analyses were performed in R version 4.0. Statistical procedures and programming codes used in the current submission are available upon request by contacting the corresponding author.
