# Supplementary material for: Treatment-resistant depression and risk of autoimmune diseases: evidence from a population-based cohort and nested case-control study
Source: Transl Psychiatry. 2023 Mar 3;13:76. doi: 10.1038/s41398-023-02383-9 (PMC9981710; doi:10.1038/s41398-023-02383-9)
Supplement: Supplementary file 1 — Supplementary legend [file 41398_2023_2383_MOESM1_ESM.pdf]

SUPPLEMENTARY MATERIALS

Supplementary Figure 1 − Schematic presentation of cohort study design

Supplementary Figure 2 − Onset distribution of autoimmune diseases among patients with and without TRD in the cohort study

Supplementary Table 1 − List of ICD-9 codes for diagnoses of depression, autoimmune diseases, and medical history
