# Supplementary material for: Treatment-resistant depression and risk of autoimmune diseases: evidence from a population-based cohort and nested case-control study
Source: Transl Psychiatry. 2023 Mar 3;13:76. doi: 10.1038/s41398-023-02383-9 (PMC9981710; doi:10.1038/s41398-023-02383-9)
Supplement: Supplementary file 2 — Supplementary materials [file 41398_2023_2383_MOESM2_ESM.pdf]

Supplementary Figure 1. Schematic presentation of cohort study design

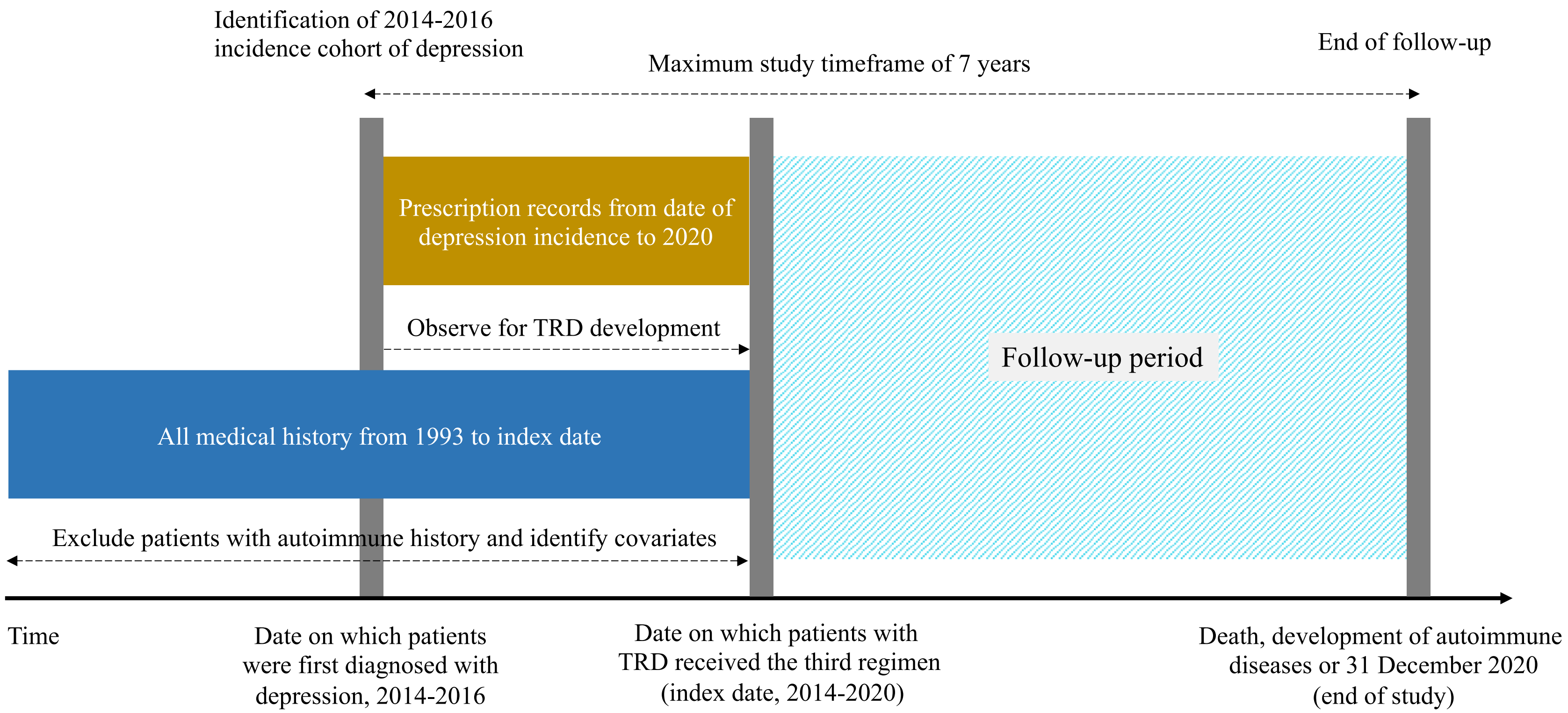

Non-TRD patients did not have the third regimen. Index dates of their matched TRD patients in the same matching stratum were assigned.
Abbreviation: TRD – Treatment-resistant depression.

Supplementary Figure 2. Onset distribution of autoimmune diseases among patients with and without TRD in the retrospective cohort study

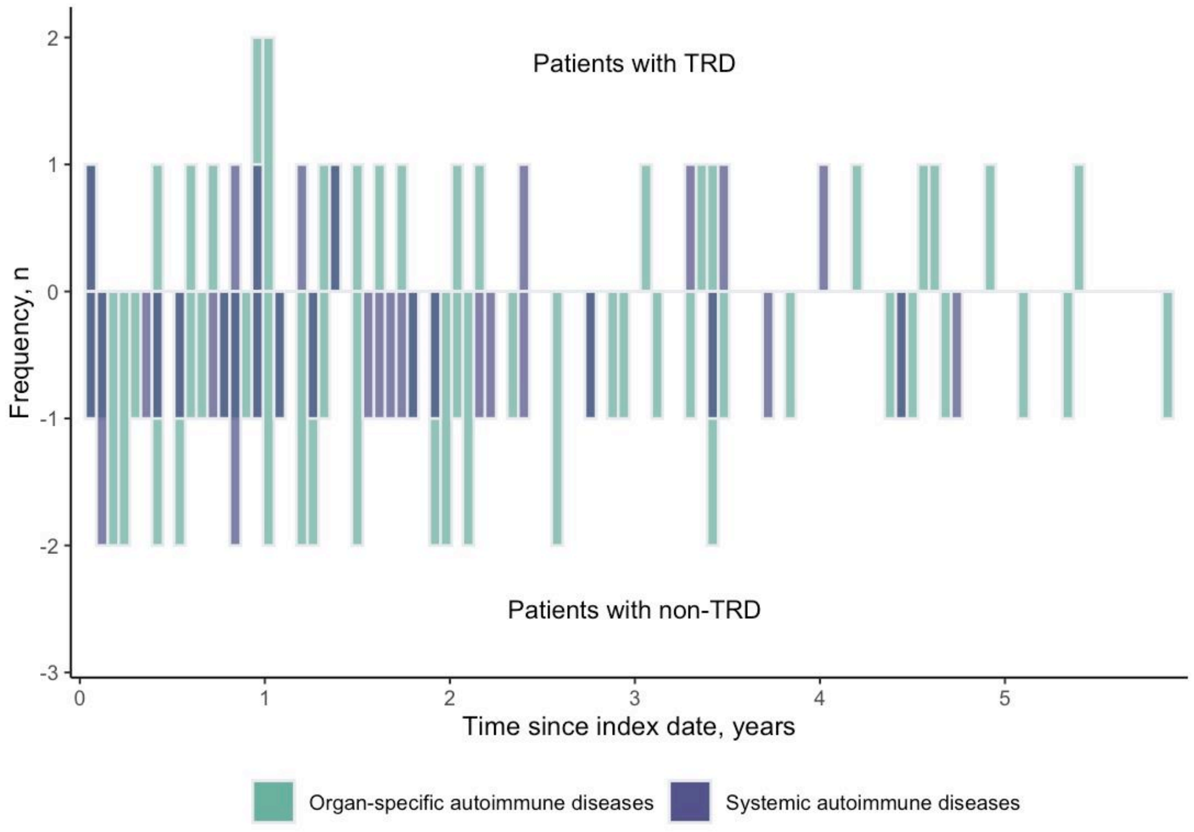

Abbreviation: TRD – Treatment-resistant depression.

Supplementary Table 1. List of ICD-9-CM codes for diagnoses of depression, autoimmune diseases, and medical history

**Diseases / Conditions**	**ICD-9-CM codes**
**Depression**[1]	
Major depressive disorder, single episode	296.2
Major depressive disorder, recurrent episode	296.3
Dysthymic disorder	300.4
Depressive disorder, not elsewhere classified	311
**Organ-specific autoimmune diseases**[2-8]	
Inflammatory bowel diseases	555-556
Ankylosing spondylitis / Spondylarthritis	720.0
Psoriasis	696
Insulin-dependent diabetes mellitus (IDDM)	250.x1, 250.x3, where x = 0-9
Hashimoto's thyroiditis	245.2
Graves' disease	242.00, 242.01
Coeliac disease	579.0
Vitiligo	709.01
Alopecia areata	704.01
Pemphigus vulgaris	694.4
Dermatitis herpetiformis	694.0
Pernicious anaemia	281.0
Immune thrombocytopenic purpura	287.31
Iridocyclitis	364.0-364.3
Pemphigoid	694.5, 694.6
**Systemic autoimmune diseases**[2,3,9]	
Systemic lupus erythematosus	710.0
Rheumatoid arthritis	714.0-714.9
Sjogren disease	710.2
Systemic sclerosis	710.1
Polymyositis/dermatomyositis	710.3-710.4
Multiple sclerosis	340
Juvenile arthritis	714.30, 714.33
**Physical disorders**	
Obesity	278
Hypertensive diseases	401-405
Non-Insulin-dependent diabetes mellitus (NIDDM)	250.x0, 250.x2, where x = 0-9
Any cardiovascular diseases<br>- Ischaemic heart disease<br>- Cerebrovascular disease<br>- Emboli and thrombosis<br>- Heart failure<br>- Arrhythmia / conduction disorder	 410-414<br>430-434, 436-438<br>415, 444, 445<br>428<br>426-427
Any tumours<br>- Non-metastatic solid tumours<br>- Metastatic solid tumours	 140-172, 174-195<br>196-199
**Psychiatric disorders**[1,10-12]	
Attention-deficit-hyperactivity disorder	314
Autism	299.0
Psychosis	297, 298
Schizophrenia	295
Epilepsy	345
Anxiety disorder	300.0, 293.84

Diseases / Conditions	ICD-9-CM codes
Personality disorder	301
Substance use disorders	292, 304.0-304.93, 305.1-305.93
Dementia	290
Bipolar disorder	296.0, 296.1, 296.4-296.8
Obsessive-compulsive disorder	300.3
Eating disorder	307.1, 307.51

Abbreviation: ICD-9-CM – International Classification of Diseases, 9th Revision, Clinical Modification.

References:

1. Chan V. K. Y., Cheung E. C. L., Chan S. S. M., Knapp M., Hayes J. F., Min F., et al. Mortality-causing mechanisms and healthcare resource utilisation of treatment-resistant depression: A six-year population-based cohort study. Lancet Reg Health West Pac. 2022:100426.

2. Li X., Tong X., Yeung W. W. Y., Kuan P., Yum S. H. H., Chui C. S. L., et al. Two-dose COVID-19 vaccination and possible arthritis flare among patients with rheumatoid arthritis in Hong Kong. Ann Rheum Dis. 2022;81(4):564-8.

3. Yang M. H. , Huang J. Y., Chen S. L., Wei J. C. Association of Interstitial Cystitis/Bladder Pain Syndrome: A Nationwide Population-Based Study. J Clin Med. 2021;10(23):5669.

4. Chen M. H., Tsai S. J., Bai Y. M., Huang K. L., Su T. P., Chen T. J., et al. Type 1 diabetes mellitus and risks of major psychiatric disorders: A nationwide population-based cohort study. Diabetes Matab. 2022;48(1):101319.

5. Sheth V. M., Guo Y., Qureshi A. A. Comorbidities associated with vitiligo: a ten-year retrospective study. Dermatology. 2013;227(4):311-315.

6. Hsu Y. M., Fang H. Y., Lin C. L., Shieh S. H. The Risk of Depression in Patients with Pemphigus: A Nationwide Cohort Study in Taiwan. Int J Environ Res Public Health. 2020;17(6):1983.

7. Tarantino M. D., Danese M., Klaassen R. J., Duryea J., Eisen M., Bussel J. Hospitalizations in pediatric patients with immune thrombocytopenia in the United States. Platelets. 2016;27(5):472-478.

8. Weng S. F., Jan R. L., Chang C., Wang J. J., Su S. B., Huang C. C., et al. Risk of Band Keratopathy in Patients with End-Stage Renal Disease. Sci Rep. 2016;6:28675.

9. Lauden A., Geishin A., Merzon E., Korobeinikov A., Green I., Golan-Cohen A., et al. Higher rates of allergies, autoimmune diseases and low-grade inflammation markers in treatment-resistant major depression. Brain Behav Immun Health. 2021;16:100313.

10. Ng V. W. S., Man K. K. C., Gao L., Chan E. W., Lee E. H. M., Hayes J. F., et al. Bipolar disorder prevalence and psychotropic medication utilisation in Hong Kong and the United Kingdom. Pharmacoepidemiol Drug Saf. 2021;30(11):1588-1600.

11. Tseng M. M., Tu C. Y., Hsieh S. F., Chang C. H. Rates and trends in healthcare-detected incidence of anorexia nervosa and bulimia nervosa; A national health insurance claim data study in Taiwan, 2002-2013. Int J Eat Disord. 2020;53(3):331-338.

12. Chai Y., Luo H., Wong G. H. Y., Tang J. Y. M., Lam T. C., Wong I. C. K., et al. Risk of self-harm after the diagnosis of psychiatric disorders in Hong Kong, 2000-10: a nested case-control study. Lancet Psychiatry. 2020;7(2):135-147.
